# Cutaneous sarcoidosis masquerading as chronic cutaneous lupus erythematosus - case report

**DOI:** 10.1186/s12895-016-0052-3

**Published:** 2016-09-20

**Authors:** Marjon Vatanchi, Kaivon Sobhani, Valerie T. Fisher, Jeffrey J. Meffert

**Affiliations:** 1 SUNY Downstate Medical Center, 450 Clarkson Avenue, Box 46, Brooklyn, NY 11203 USA; 2 Cancer Treatment and Research Center, Mail Code 7876, 7979 Wurzbach Road, San Antonio, TX 78229-4427 USA

**Keywords:** Sarcoidosis, Lupus, Chronic cutaneous lupus erythematosus, Discoid lupus, CCLE, DLE

## Abstract

**Background:**

Sarcoidosis is a multisystemic granulomatous disease of unknown origin. Chronic cutaneous lupus erythematosus (CCLE) is an autoimmune disease that is associated with autoantibody production and T-cell dysfunction. Cutaneous manifestations of sarcoidosis may mimic CCLE and vice versa making it difficult to reach a diagnosis clinically.

**Case presentation:**

We present a case of a 57-year-old woman with long-standing sarcoidosis who presented to clinic with diffuse painful plaques that were very distinct and suggestive of CCLE. She had a family history of both sarcoidosis and CCLE. The patient was immediately started on topical corticosteroids and oral hydroxychloroquine. Skin biopsy and the absence of direct immunofluorescence confirmed a skin manifestation of her previously diagnosed sarcoidosis, despite the clinical morphology favoring classic CCLE.

**Conclusion:**

Sarcoidosis may have diverse manifestations and may mimic other disease processes. A detailed history along with a low threshold for biopsy is important for determining a diagnosis.

## Background

Sarcoidosis is a multisystemic granulomatous disease of unknown origin. Cutaneous manifestations are present in 30 % of patients with sarcoidosis and may be the first presenting sign [1]. On physical exam alone, it may be difficult to distinguish between cutaneous manifestations of sarcoidosis and CCLE. Fortunately, these conditions have distinct findings on histology that make diagnosis easy with a skin biopsy. Furthermore, ancillary tests can also help facilitate the process, these include direct immunofluorescence, laboratory work-up, and visceral imaging.

## Case presentation

A 57-year-old African-American female presented with hyperpigmented plaques on the face, scalp, arms, hands, buttocks, legs, and feet. She has had similar lesions over the past fifteen years and described them as burning, itchy, and painful.

**Fig. 1:**
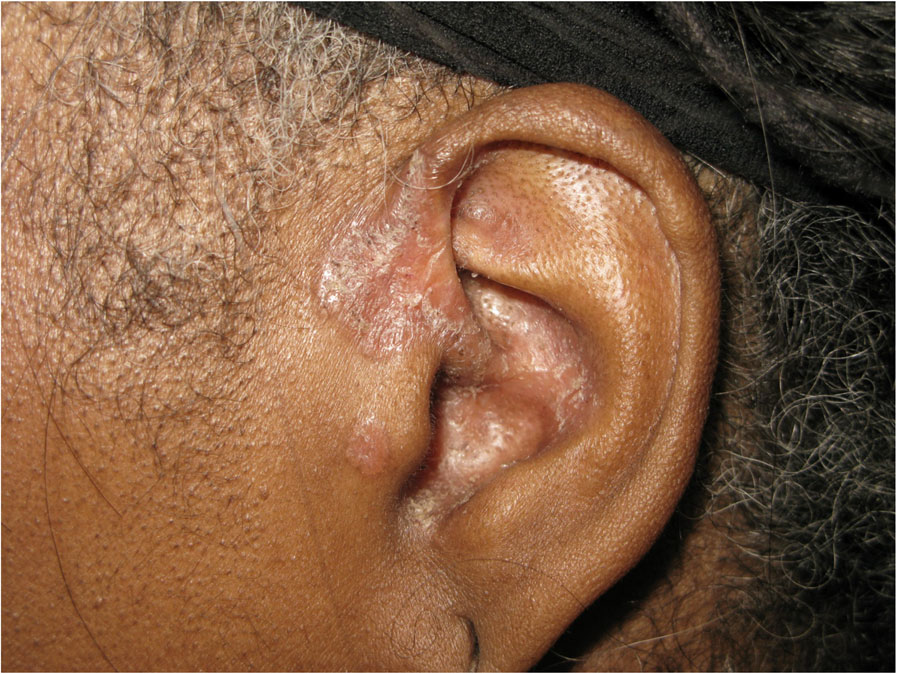
Left ear displays pink atophic plaques with adherent scale and follicular plugging in the conchal bowl and preauricular region

**Fig. 2:**
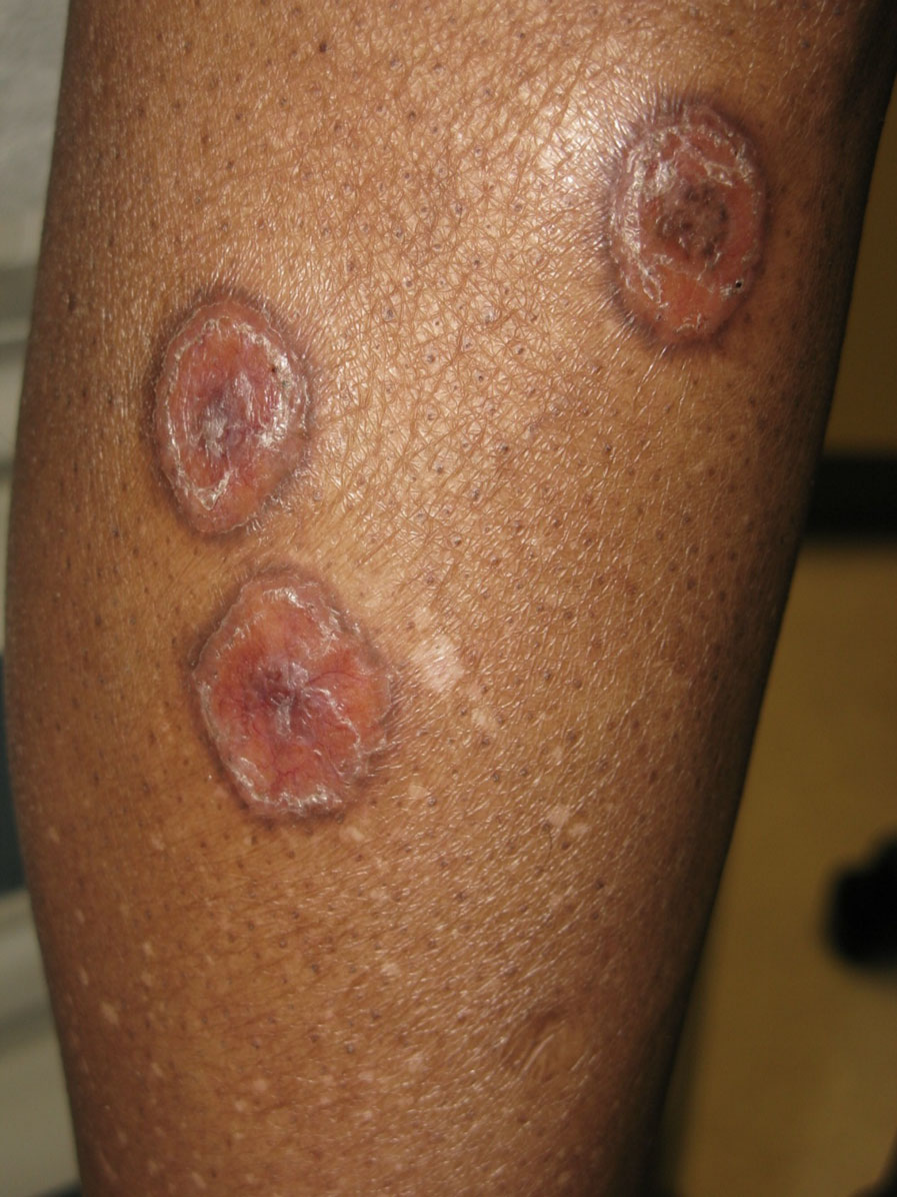
The anterior lower leg exhibits multiple dyschromic annular plaques 1–4 cm in size with scaling and hyperpigmentaed rolled borders on the periphery

**Fig. 3:**
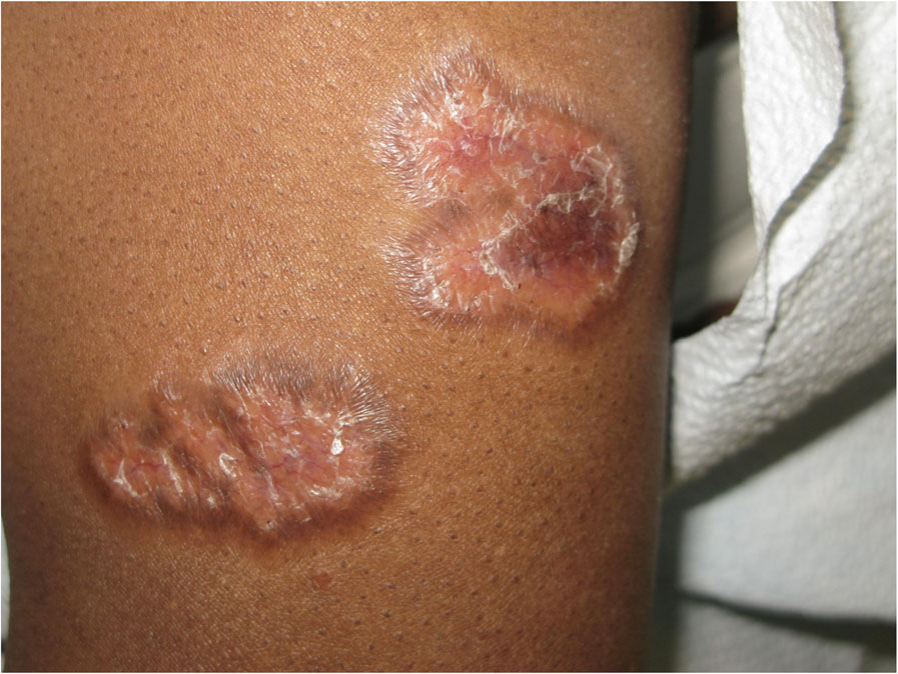
The upper arm displays indurated raised plaques with dyspigmentation and scaling

There were dermal papules on the perinasal and periocular areas. The ears displayed plaques with adherent scale and follicular plugging in both conchal bowls and preauricular regions (Fig. 1). The anterior hard palate had diffuse erythema and a small ulceration distally. The dorsal arms and anterior lower legs had multiple 1-4 cm dyschromic annular plaques exclusively in sun-exposed areas with central scaling and hyperpigmented, slightly rolled borders (Fig. 2). Induration was present in most of the lesions (Fig. 3).

She had been diagnosed with systemic sarcoidosis twelve years prior as well as hypertension and congestive heart failure eight years earlier. Her medications included aspirin, metoprolol, and ibuprofen as needed for pain. The patient reported a history of lupus (unknown subtype) in her maternal aunt and sarcoidosis in her maternal sister and two uncles.

Laboratory studies included complete blood count with differential, complete metabolic panel, brain natriuretic peptide, erythrocyte sedimentation rate (ESR), and angiotensin-converting-factor (ACE). The only abnormal values were an ESR of 41 mm/h (reference range 0-30 mm/h) and ACE of 73 nmol/mL/min (reference range 9–67 nmol/mL/min).

Given the patient’s history of long-standing lesions; photodistribution; and the presentation of plaques with dyschromia, atrophy, follicular plugging, and raised borders, a diagnosis of CCLE was favored. While the patient had a past medical history of sarcoidosis, her current lesions appeared classic for CCLE and both conditions were present in her family history. Given the information and clinical presentation, a biopsy proved necessary as a purely clinical diagnosis was not adequate.

**Fig. 4:**
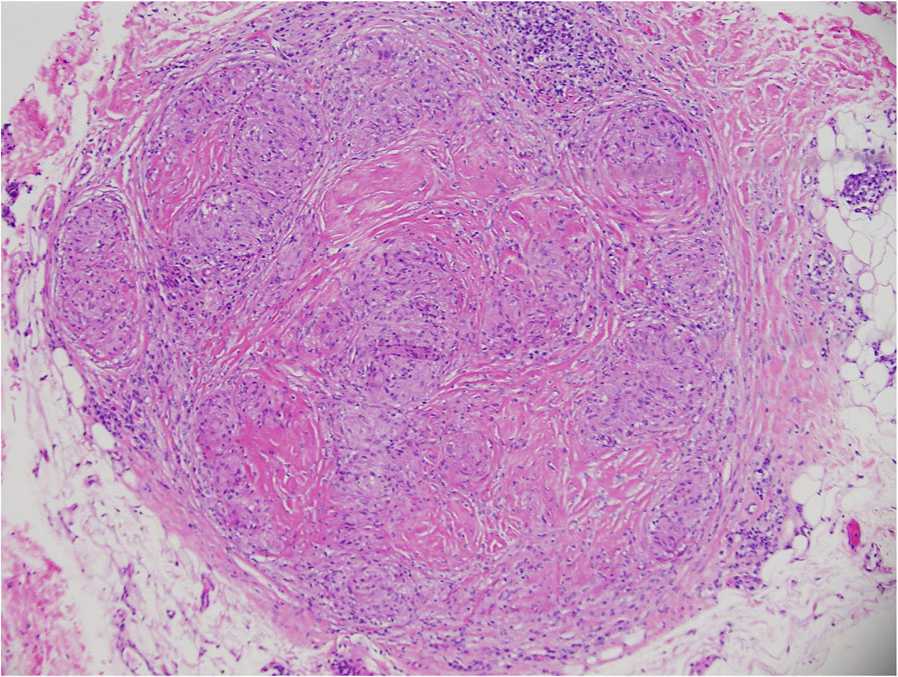
Punch biopsy taken from a plaque on the left arm. There are many non-caseating (naked) granulomas with few scattered lymphocytes (hemotoxylin and eosin stain, magnification × 10)

**Fig. 5:**
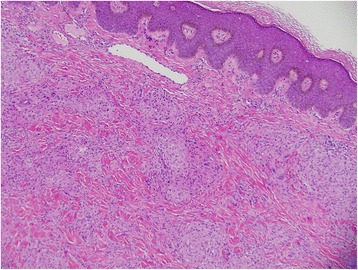
Histology reveals more non-caseating granulomas with a normal dermal-epidermal junction and no hyperkeratosis (hemotoxylin and eosin stain, magnification × 10)

Two punch biopsies were taken from a plaque on the left arm and sent for histopathology and direct immunofluorescence. Pathology exhibited non-caseating granulomas (Figs. 4 and 5) with multinucleated giant cells and a few lymphocytes at the periphery typical for sarcoidosis. Stains for acid fast bacilli and fungi were negative. Direct immunofluorescence studies were negative. While the cutaneous morphology strongly favored CCLE, there were no histologic findings to support this diagnosis. The patient remained on topical corticosteroids and oral hydroxychloroquine. On one-month and three-month follow-up visits, she had slow but significant improvement in her cutaneous symptoms.

## Discussion

Our patient represents a case of sarcoidosis with skin manifestations that closely imitate those of CCLE. The lesions seen in sarcoidosis are divided into specific and nonspecific subgroups based on histologic findings. Specific lesions occur in 9-15 % of patients and exhibit noncaseating granulomas [2]. The most common nonspecific cutaneous finding of sarcoidosis is erythema nodosum [1]. Our patient exhibited both specific and nonspecific lesions, displaying noncaseating granulomas on pathology and erythema nodosum on physical examination.

In sarcoidosis, noncaseating granulomas can be found in tissues and organs such as the skin, lungs, and lymph nodes. These granulomas consist of aggregates of epithelioid histiocytes, giant cells, and mature macrophages [3]. They are surrounded by sparse lymphocytic infiltrates composed primarily of CD4+ T-cell lymphocytes and few CD8+ lymphocytes [4]. Special stains and cultures to rule out acid fast bacilli and fungi are essential as sarcoidal granulomas have no unique histologic features to differentiate them from other noncaseating granulomas [5].

Cutaneous manifestations of CCLE include erythematous to violaceous scaly plaques with prominent follicular plugging that often results in scarring and atrophy. They are found most commonly on the face, scalp, ears, and less commonly on the mucosal surfaces, conjunctivae, and genital mucosa. Plaques usually appear in a photodistributed pattern, although not always necessary. Skin morphology includes dyspigmentation in longstanding lesions with hypopigmentation in the central region and hyperpigmentation at the periphery [6]. Hypertrophic CCLE is a variant in which lesions exhibit thick scaling, follicular plugging, and atrophic scarring in chronic lesions.

Histologically, CCLE plaques are found to have inflammatory infiltrates in the superficial and deep dermis as well as the surrounding adnexal structures. Keratinocyte damage and hyperkeratosis are present in the epidermis with possible prominence of amorphous and eosinophilic colloid bodies in the epidermis and/or upper dermis. Melanin deposits within macrophages are located in the dermis. Lymphohistiocytic cellular infiltrates that are often pronounced are located in the dermis at perivascular and periadnexal locations. If histopathology is equivocal for CCLE, direct immunofluorescence may be helpful. In cutaneous lupus, antibody deposition is seen at the dermal-epidermal junction and around hair follicles [7]. While many ancillary tests can be performed such as autoantibodies to dsDNA, Smith and anti-nuclear antibodies, it is histology and direct immunofluorescence that become essential for accurate diagnosis of CCLE.

## Conclusion

Without a biopsy, one might be quick to diagnose this individual with known sarcoidosis with superimposed CCLE based on the clinical morphology. Her plaques had all the characteristic cutaneous findings including dyschromia, atrophy, adherent scales, central hypopigmentation, and hyperpigmentation at the periphery. However, histology did not show follicular plugging or lymphohistiocytic cellular infiltrates. Immunofluorescence studies were also negative. Histology was pathognomonic for sarcoidosis exhibiting non-caseating granulomas, giant cells, and a lymphocytic infiltrate. In the end, strong clinical inclination was not enough and a biopsy was the necessary factor in determining a definitive diagnosis.

## Abbreviations

ACE: Angiotensin converting enzyme
CCLE: Chronic cutaneous lupus erythematosus
DLE: Discoid lupus erythematous
ESR: Erythrocyte sedimentation rate
